# AKT1 Phosphorylates FDX1 to Promote Cuproptosis Resistance in Triple‐Negative Breast Cancer

**DOI:** 10.1002/advs.202408106

**Published:** 2025-02-20

**Authors:** Zicheng Sun, Huazhen Xu, Guanming Lu, Ciqiu Yang, Xinya Gao, Jing Zhang, Xin Liu, Yongcheng Chen, Kun Wang, Jianping Guo, Jie Li

**Affiliations:** ^1^ Department of Breast and Thyroid Surgery Guangzhou Women and Children's Medical Center Guangzhou Medical University Guangzhou Guangdong 510000 China; ^2^ Department of Breast and Thyroid Surgery Affiliated Hospital of Youjiang Medical University for Nationalities and Key Laboratory of Molecular Pathology in Tumors of Guangxi Guangxi 533000 China; ^3^ Department of Breast Cancer Guangdong Provincial People's Hospital (Guangdong Academy of Medical Sciences) Southern Medical University Guangzhou Guangdong 510000 China; ^4^ Institute of Precision Medicine The First Affiliated Hospital Sun Yat‐sen University Guangzhou Guangdong 510000 China

**Keywords:** AKT1, breast cancer, cuproptosis, FDX1, metabolic reprogramming

## Abstract

Cuproptosis, a recently defined copper‐dependent cell death pathway, remains largely unexplored in tumor therapies, particularly in breast cancer. This study demonstrates that triple‐negative breast cancer (TNBC) bears a relatively elevated copper levels and exhibits resistance to cuproptosis. Mechanistically, copper activates the AKT signaling pathway, which inhibits ferredoxin‐1 (FDX1), a key regulator of cuproptosis. AKT1‐mediated FDX1 phosphorylation not only abrogates FDX1‐induced cuproptosis and aerobic respiration but also promotes glycolysis. Consequently, the combination of AKT1 inhibitors and the copper ionophores synergistically alleviate TNBC tumorigenesis both in vitro and in vivo. In summary, the findings reveal a crucial mechanism underlying TNBC resistance to cuproptosis and suggest a potential therapeutic approach for TNBC.

## Graphical abstract

This study demonstrates that copper activates the AKT signaling pathway, which inhibits ferredoxin‐1 (FDX1), a key regulator of cuproptosis. AKT1‐mediated FDX1 phosphorylation not only abrogates FDX1‐induced cuproptosis and aerobic respiration but also promotes glycolysis. Consequently, the combination of AKT1 inhibitors and the copper ionophores synergistically alleviates TNBC tumorigenesis both in vitro and in vivo.

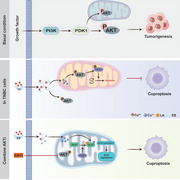

## 1 Introduction

Copper, an essential trace element, plays a crucial role in various physiological processes, including mitochondrial respiration, antioxidant defense, redox signaling, and kinase signaling.^[^ ^1^, ^2^, ^3^ ^]^ Copper concentrations are typically elevated in tumor tissues and blood, which indicates a potential risk factor and prognostic indicator.^[^ ^4^, ^5^, ^6^, ^7^, ^8^, ^9^, ^10^, ^11^ ^]^ Copper metabolism plays dual roles in tumorigenesis and cancer therapy. Cuproplasia refers to the role of copper in promoting cell proliferation and growth. Copper directly activates oncogenic signaling pathways, leading to the expression of key proteins such as HIF1α, GPER, and VEGF in breast cancer, and promoting tumorigenesis and angiogenesis.^[^ ^4^, ^12^ ^]^ Recent studies have highlighted the role of copper in the immune regulation of breast cancer, including the upregulation of PD‐L1 expression via activation of the STAT3 and EGFR signaling pathways, which enable immune evasion.^[^ ^4^, ^13^ ^]^

Paradoxically, copper also facilitates cell death through various mechanisms, including apoptosis, pyroptosis, ferroptosis, and more recently, cuproptosis.^[^ ^4^, ^5^ ^]^ Copper‐induced reactive oxygen species (ROS) can activate NLRP3 inflammasomes, leading to pyroptosis.^[^ ^5^, ^14^, ^15^, ^16^ ^]^ Increasing evidence suggests that copper affects ferroptosis.^[^ ^17^ ^]^ Copper chelators or complexes, such as disulfiram and elesclomol, disrupt mitochondrial homeostasis, resulting in oxidative stress and ferroptosis.^[^ ^18^, ^19^, ^20^ ^]^ Cuproptosis, a newly identified form of cell death, is characterized by the binding of copper to lipoylated proteins in the tricarboxylic acid (TCA) cycle, causing abnormal protein aggregation and subsequent cell death.^[^ ^21^ ^]^ Despite the elevated copper levels, TNBC cells exhibit resistance to cuproptosis. Therefore, the regulation and therapeutic targeting of this novel death mechanism in tumorigenesis warrant further investigation. FDX1 is a crucial regulator of cuproptosis and induces the conversion of Cu(II) to Cu(I), which promotes mitochondrial proteotoxic stress via protein lipoylation and subsequent dihydrolipoamide S‐acetyltransferase (DLAT) aggregation.^[^ ^21^, ^22^, ^23^ ^]^ Thus, targeting FDX1 profoundly affects cellular sensitivity to cuproptosis. Lactylation of METTL16 at residue K229 promotes FDX1 accumulation through m^6^A modification of FDX1 mRNA, followed by cuproptosis.^[^ ^24^ ^]^ Conversely, p38‐MAPK activation leads to the phosphorylation and nuclear translocation of FOXO3, which inhibits FDX1 expression, thereby suppressing cuproptosis.^[^ ^25^ ^]^

In our study, we discovered that excessive copper in TNBC activates AKT1, which correlates with cuproptosis resistance. Specifically, AKT1 inhibited the protein lipoylation by phosphorylating FDX1 while simultaneously regulating tumor metabolic reprogramming, ultimately suppressing cuproptosis. These findings revealed a crucial mechanism underlying TNBC resistance to cuproptosis and suggested a potential therapeutic approach for TNBC.

## 2 Results

### 2.1 Triple Negative Breast Cancer is Resistant to Cuproptosis

**Figure 1:**
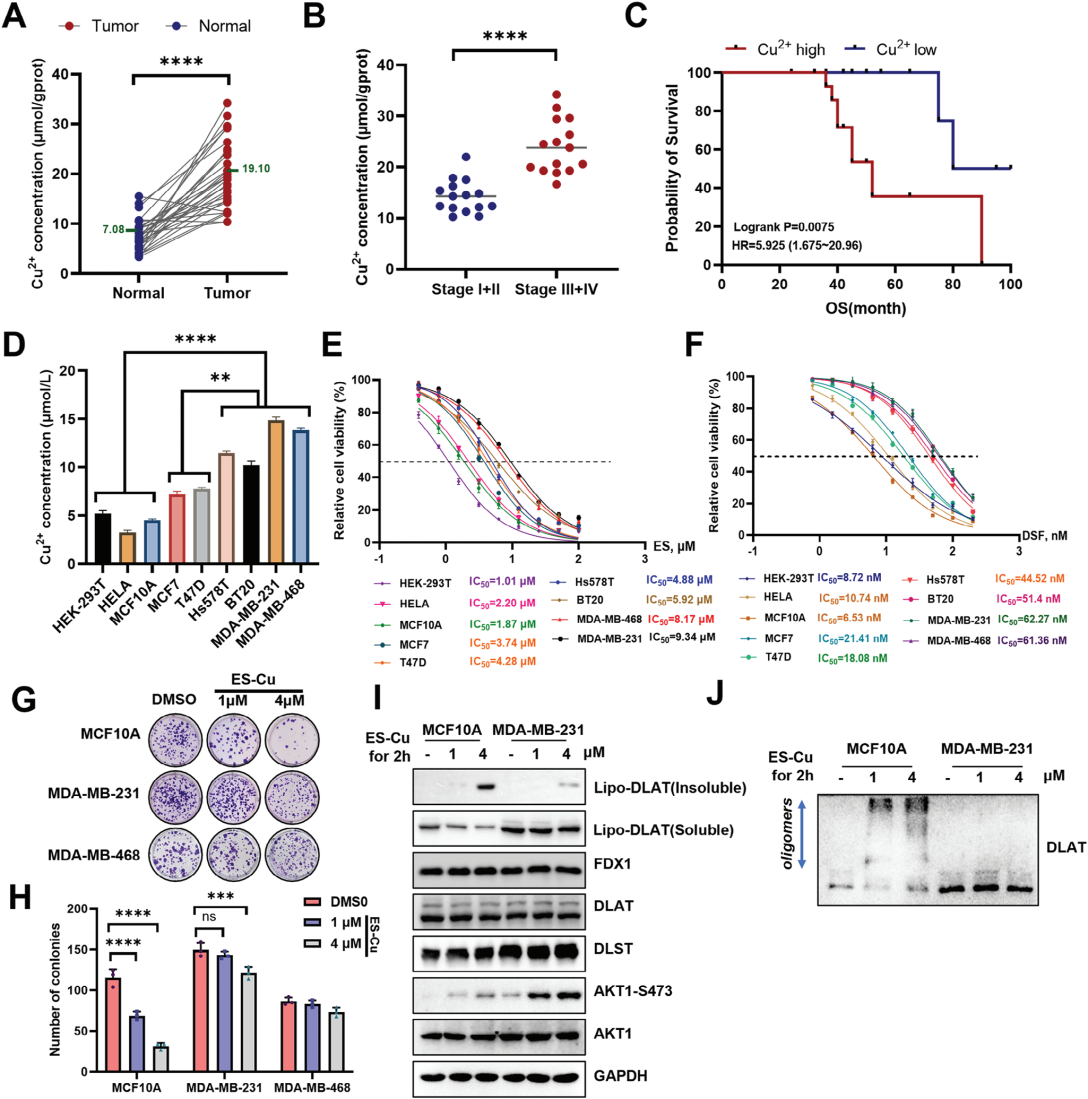
Triple negative breast cancer exhibits resistance to cuproptosis. A) Paired line scatter plot showing Cu concentration in TNBC tissues (right, *n* = 30) and adjacent normal tissues (left, *n* = 30). Copper concentrations were significantly elevated in TNBC tissues compared to adjacent normal tissues. The means were indicated in purple font for clarity. B) Box plot illustrating Cu concentrations in patients with Stage I + II (*n* = 15) and Stage III + IV (*n* = 15) TNBC. C) Kaplan–Meier analysis depicting the association between Cu concentrations and overall survival in TNBC patients. D) Box plot showing Cu concentrations in normal breast cells and TNBC cells, *n* = 3. E, F) The IC_50_ values of elesclomol‐Cu or disulfiram‐Cu were determined for normal breast cells and TNBC cells, *n* = 3. G,H) Colonies were visualized by crystal violet staining (G), and the bar graphs (H) quantified the colony counts, *n* = 3. I) Lipoylated DLAT expression was analyzed in MCF‐10A and MDA‐MB‐231 cells after a 2 h pulse treatment with elesclomol‐Cu. J) The DLAT oligomerization was analyzed in MCF‐10A and MDA‐MB‐231 cell treated with elesclomol‐Cu for 2 h pulse. The culture medium was supplemented with 1 µm CuCl_2_ for experiments shown in panels (E–J). All data are presented as the mean±SD (n ≥ 3). The *p*‐value in panels (A) and (B) was calculated using a paired *t*‐test. The *p*‐value in panel (D) was calculated using *one‐way ANOVA*. The *p*‐value in panel (H) was determined using *two‐way ANOVA*. ^**^ *p* < 0.01; ^***^ *p* < 0.001; ^****^ *p* < 0.0001.

Copper levels are elevated in tumors and play a potential role in tumorigenesis, including breast cancer.^[^ ^26^, ^27^ ^]^ As a major copper transporter, copper transporter 1 (CTR1) is tightly regulated at the transcriptional level by HIF1α and C‐myc and at the post‐translational modification level by Nedd4‐1 and AMPK, leading to copper elevation in tumors.^[^ ^28^, ^29^ ^]^ Consistent with these findings, copper levels were significantly elevated in TNBC tissues compared to adjacent tissues (**Figure** **1**A) and were positively correlated with advanced stages and poor prognosis in patients (Figure 1B,C). TNBC cells exhibited higher copper levels than that in normal breast and LuminalA breast cancer cells; similar findings were observed in breast cell lines (Figure 1D). Based on previous findings that elesclomol (ES) and disulfiram (DSF) deliver copper into cells and subsequently induce cuproptosis, we hypothesized that elevated copper levels in TNBC enhance the efficacy of drug‐induced cuproptosis.^[^ ^27^, ^30^ ^]^ However, cancer cells with higher copper levels exhibited resistance to cuproptosis, as indicated by the cell viability and colony formation assays (Figure 1E–H). To confirm that the reduction in cell viability was due to cuproptosis rather than other cell death pathways, we assessed the insoluble lipo‐DLAT protein levels and oligomerization. The results demonstrated that elesclomol‐induced cuproptosis occurred more robustly in normal cells with lower copper levels, however, not in TNBC cells (Figure 1I,J). Collectively, these findings indicated that TNBC cells elevated copper levels and exhibited resistance to elesclomol‐induced cuproptosis.

### 2.2 Phosphoproteomic Profiling Identified AKT1 as a Key Gene for Cuproptosis Sensitivity

**Figure 2:**
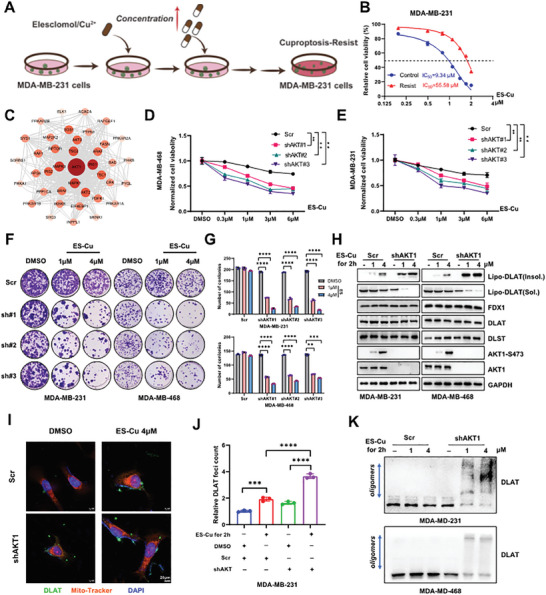
AKT1 contributes to resistance to cuproptosis. A) Schematic diagram depicting the workflow for establishing cuprotosis‐resistant MDA‐MB‐231 cells. B) The IC_50_ value of elesclomol‐Cu was determined in wild‐type (WT) and resistant MDA‐MB‐231 cells, *n* = 3. C) The key differential proteins were mapped onto a PPI network. D, E) MDA‐MB‐468 or MDA‐MB‐231 cells transduced with lentiviral shRNAs targeting AKT1 were treated with DMSO or elesclomol‐Cu and subjected to CCK‐8 assays to evaluate cell viability, *n* = 3. F) Cells generated in (D, E) were treated with DMSO or elesclomol‐Cu and subjected to colony formation assays. G) Bar graphs provided a statistical evaluation of colony counts for MDA‐MB‐231 cells (top panel) and MDA‐MB‐468 cells (bottom panel), *n* = 3. H) The lipoylated DLAT expression was analyzed in cells generated in (D, E) following a 2 h pulse treatment with elesclomol‐Cu. I) DLAT foci in cells generated in (E) were visualized through immunofluorescence staining (DLAT: green, Mitotracker: red, DAPI: blue). J) DLAT foci were measured and quantified by ImageJ, *n* = 3. K) DLAT oligomerization was analyzed in cells generated in (D, E) following a 2 h pulse treatment with elesclomol‐Cu. The culture medium was supplemented with 1 µm CuCl_2_ for experiments shown in all panels. All data are presented as the mean ± SD (n ≥ 3). The *p*‐value in panels (D), (E), and (G) was calculated using *two‐way ANOVA*. The *p*‐value in panel (J) was calculated using o*ne‐way ANOVA*. ^**^ *p* < 0.01; ^***^ *p* < 0.001; ^****^ *p* < 0.0001.

To explore the key factors modulating the sensitivity to cuproptosis, we initially generated cuproptosis‐resistant TNBC cells. As a result, the cuproptosis‐resistant MDA‐MB‐231 cells exhibited a significantly increased IC_50_ compared to WT cells (55.58 vs. 9.34 µm) under chronic stimulation of copper‐elesclomol (**Figure** **2**A,B; Figure S1A,B, Supporting Information). Consistently, cuproptosis‐resistant cells showed lower levels of lipoylated DLAT, and reduced DLAT oligomerization, and no increase in DLAT foci upon elesclomol treatment (Figure S1C–F, Supporting Information).

Given the crucial role of copper in promoting protein phosphorylation to maintain copper homeostasis,^[^ ^7^, ^28^ ^]^ we speculated that abnormal phosphorylation might influence cuproptosis sensitivity. Further, we performed phosphoproteomic profiling to compare resistant and intact MDA‐MB‐231 cells. In total, 7372 proteins and 13831 phosphosites were quantified, of which 2170 proteins were upregulated, and 1344 proteins were downregulated in the resistant cells compared to the intact cells, using an FDR cutoff of 1.2. KEGG enrichment analysis of upregulated differentially phosphorylated proteins in the cuproptosis‐resistant cells revealed significant enrichment in several pathways, including the insulin signaling pathway (Figure S1G, Supporting Information). Furthermore, domain enrichment analysis revealed that the upregulated phosphosites were enriched in AGC kinase motifs (RxxS/T) (Figure S1H, Supporting Information). As multiple AGC kinases, such as AKT, PDK1, S6K, and SGK, are key factors in the insulin signaling pathway, we subsequently conducted a protein‐protein interaction analysis, which revealed that AKT1 was the most pivotal protein among them (Figure 2C). Based on the close connection between copper and AKT,^[^ ^29^ ^]^ we observed that copper ions combined with elesclomol promoted AKT1 phosphorylation (Figure S1I, Supporting Information), possibly due to enhanced PDK1 interaction with AKT1 (Figure S1J, Supporting Information). Elesclomol and copper ions failed to activate AKT1 in *PDK1*‐deficient TNBC cells (Figure S1K, Supporting Information). In addition, the activation of AKT1 oncogenic signaling in cuproptosis‐resistant TNBC cells has been reported (Figure S1L, Supporting Information). Therefore, we hypothesized that AKT1 was a potential mediator of cuproptosis resistance.

To further explore the relationship between AKT1 and cuproptosis resistance, we analyzed the effect of *AKT1* depletion on cell viability following treatment with elesclomol or disulfiram combined with copper ions. The results demonstrated that *AKT1* knockdown significantly sensitized TNBC cells to cuproptosis, as assessed by cell viability and colony formation assays (Figure 2D–G; Figure S2A–C, Supporting Information). Cuproptosis is dependent on the FDX1‐mediated lipoylation pathway. In other words, copper directly binds to lipoylated DLAT, inducing DLAT oligomer formation and insoluble DLAT, ultimately leading to cell death. Consistently, both pharmacological and genetic repression of AKT1 led to a marked increase in insoluble lipoylated DLAT expression, decreased soluble lipoylated DLAT expression (Figure 2H; Figure S2D,E, Supporting Information), and increased DLAT oligomerization and DLAT foci formation (Figure 2I–K; Figure S2F–H, Supporting Information). Consistent with this, the AKT1 inhibitor, MK2206, promoted cuproptosis in vivo, as evidenced by increased insoluble lipoylated DLAT expression (Figure 6I). Conversely, activation of AKT1 by insulin significantly reduced insoluble lipoylated expression and oligomerization of DLAT (Figure S2I–K, Supporting Information). Collectively, these findings suggest that AKT1 plays a pivotal role in the negative regulation of cuproptosis sensitivity.

### 2.3 AKT1 Directly Phosphorylates FDX1

**Figure 3:**
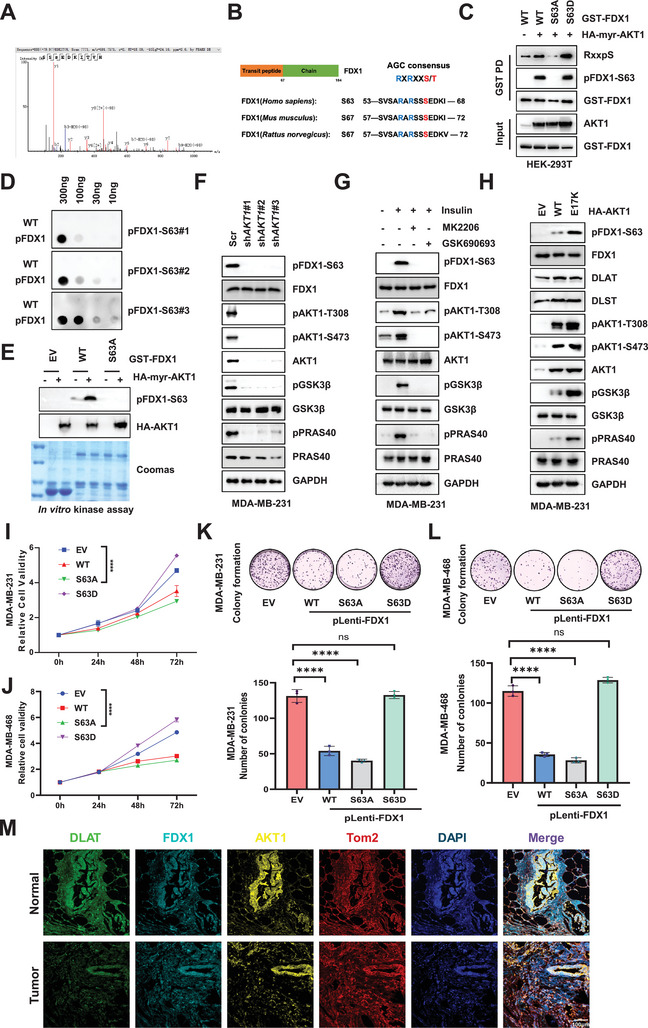
AKT1 phosphorylates FDX1 at Ser63. A) GST‐pulldown products from HEK‐293T cells transfected with GST‐FDX1 and HA‐myr‐AKT1 were analyzed by mass spectrometry. B) Schematic representation of the evolutionarily conserved putative AKT1 kinase phosphorylation consensus sequence in FDX1. C) IB analysis of WCL and GST‐pulldown products derived from HEK‐293T cells transfected with GST‐FDX1 WT or variants and HA‐myr‐AKT1. D) IB analysis of the specified synthetic peptides immobilized on a nitrocellulose membrane. E) In vitro kinase assays were conducted with the purified AKT1 proteins from HEK‐293T cells transfected with HA‐myr‐AKT1 as the kinase source, and bacterially purified GST‐FDX1 as substrate. F) IB analysis of WCL derived from MDA‐MB‐231 cells infected with the lentiviral shRNA targeting *AKT1*. G) IB analysis of WCL derived from MDA‐MB‐231 cells. Cells were serum‐starved for 24 h and treated with or without AKT inhibitors (MK2206, 10 µm; GSK690693, 10 µm) for 1 h, and then subjected to insulin (100 nM) stimulation for 30 min before harvesting. H) IB analysis of WCL derived from MDA‐MB‐231 cells stably overexpressing HA‐AKT1 WT or variants. I, J) MDA‐MB‐231 and MDA‐MB‐468 cells stably overexpressing FDX1 WT or variants were subjected to CCK‐8 assays to assess cell viability, *n* = 3. K) Cells generated in (I) were subjected to colony formation assays, and the relative colony numbers were quantified (bottom panel), *n* = 3. L) Cells generated in (J) were subject to colony formation assays, and the relative colony numbers were quantified (bottom panel), *n* = 3. M) DLAT, FDX1, and AKT1 staining were visualized through immunofluorescence in TNBC and adjacent normal tissues. All data are presented as the mean ± SD (n ≥ 3). The *p*‐value in panels (I) and (J) was calculated using two*‐way ANOVA*. The *p*‐value in panel (K) and (L) was calculated using o*ne‐way ANOVA*. ^***^ *p* < 0.001; ^****^ *p* < 0.0001; ns, not significance.

To investigate the underlying mechanism by which AKT1 inhibits cuproptosis, we performed MS‐based proteomic analyses and found that AKT1 interacted with FDX1 (**Figure** **3**A), a key regulator of cuproptosis. This interaction was further confirmed in both the ectopic expression system and the endogenous setting in TNBC cells and tissues (Figure 3M; Figure S3A,B, Supporting Information). Consistently, in vitro pulldown assays demonstrated direct binding between FDX1 and AKT1 (Figure S3C, Supporting Information), while confocal microscopy confirmed their co‐localization (Figure S3F, Supporting Information). Moreover, we identified the N‐terminal domain of FDX1 as a crucial component mediating its interaction with AKT1 (Figure S3D,E, Supporting Information).

Given that AKT1 functions as a kinase that phosphorylates its substrates, we investigated whether it could directly phosphorylate FDX1. MS analysis identified several potential phosphorylation sites on FDX1, with serine 63 (S63) drawing particular attention due to its location within the conserved AGC kinase phosphorylation motif (RxRxxS/T) (Figure 3A,B). Screening of a panel of AGC kinases, including constitutively active forms of AKT1 (myr‐AKT1), AKT2 (myr‐AKT2), S6K1 (S6K1‐R3A), and SGK1 (SGK1‐Δ60), revealed that only AKT1 significantly promoted FDX1 phosphorylation, as detected by a pan‐AKT substrate phosphorylation antibody (Figure S4A, Supporting Information).

Further experiments confirmed that AKT1‐induced FDX1 phosphorylation of FDX1 predominantly occurred at its N‐terminal region in vitro and in vivo (Figure S4B,C, Supporting Information). Notably, mimicking the non‐phosphorylated mutant FDX1‐S63A abolished AKT1‐mediated phosphorylation (Figure 3C). Notably, we successfully generated and validated phosphorylation‐specific antibodies that recognized FDX1‐S63 (Figure 3D). Consistent with this finding, in vitro kinase assays coupled with MS analysis confirmed that the S63A mutation abolished AKT1‐mediated FDX1 phosphorylation (Figure 3A,E). Additionally, insulin stimulation enhanced FDX1‐S63 phosphorylation in TNBC cells (Figure 3G), whereas the patient‐derived AKT1‐activating mutation (E17K) significantly accelerated this phosphorylation (Figure 3H). Conversely, AKT1 knockdown or treatment with AKT1 inhibitors significantly reduced FDX1‐S63 phosphorylation (Figure 3F,G). The phosphorylation level of FDX1‐S63 highly correlated with AKT1 activity in both breast cancer cell lines and tumor tissues (Figure S5C–E, Supporting Information). Taken together, these findings suggested that AKT1 can directly phosphorylate FDX1 at Ser63.

### 2.4 AKT1‐Mediated FDX1 Phosphorylation Promotes TNBC Tumor Growth by Inhibiting Cuproptosis

**Figure 4:**
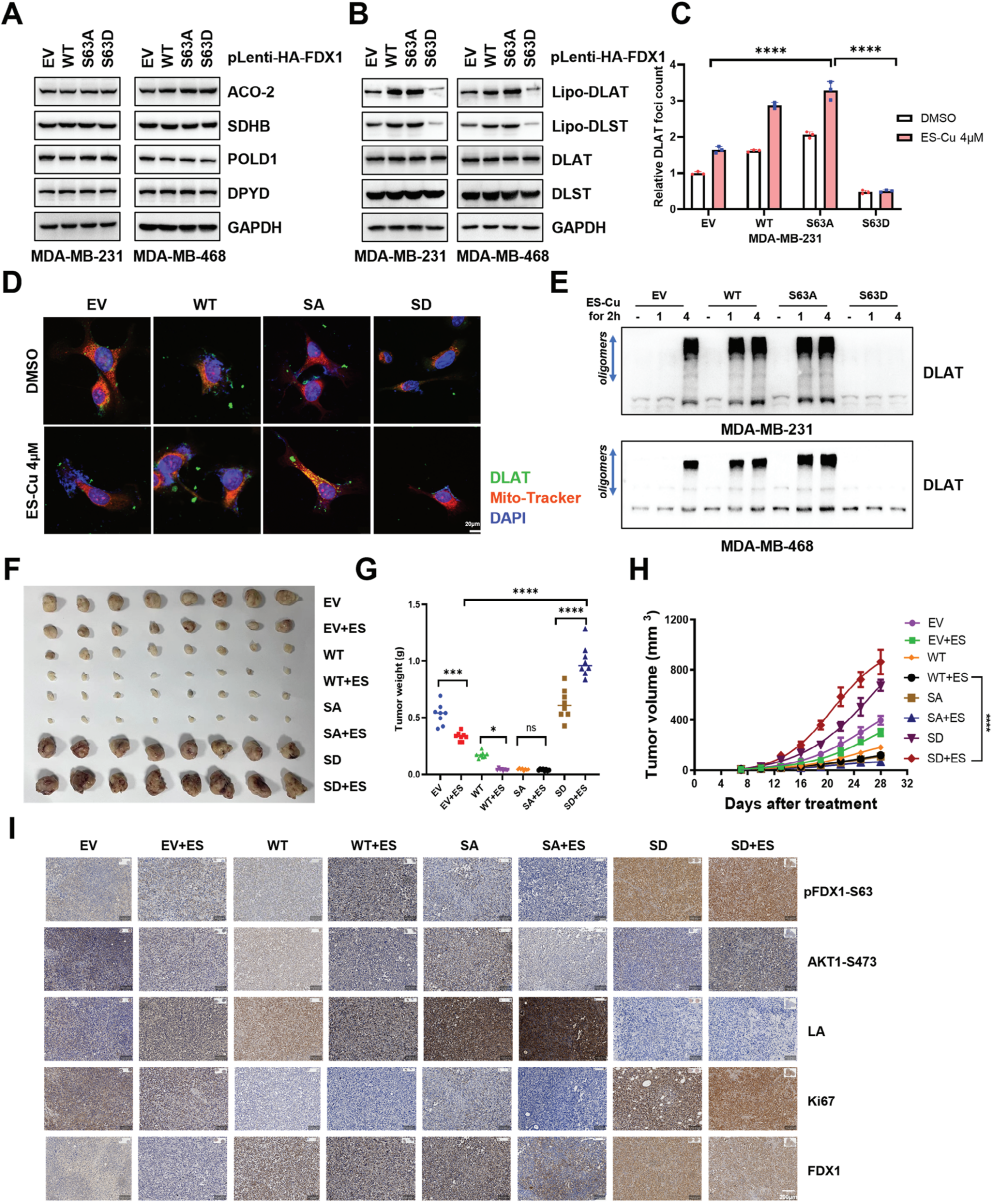
AKT1‐mediated FDX1 phosphorylation inhibits cuproptosis. A) MDA‐MB‐231 and MDA‐MB‐468 cells stably overexpressing FDX1 WT or its variants were subjected to IB analysis to assess effect on Fe‐S cluster biogenesis. B) Cells generated in (A) were subjected to IB analysis to assess effect on the lipoylation pathway. C) DLAT foci were measured and quantified using ImageJ, *n* = 3. D) DLAT foci in cells generated in (A) were visualized through immunofluorescence staining (DLAT: green, Mitotracker: red, DAPI: blue). E) DLAT oligomerization was analyzed in cells generated in (A) following a 2 h pulse treatment with elesclomol. F,G,H) Cells generated in (A) were subjected to mouse xenograft assays with vehicle or elesclomol (20 mg kg^−1^), administered three times a week for 21 days. Dissected tumors were weighed (G), and tumor sizes were monitored (H), *n* = 8. I) IHC staining was performed on tissues derived from dissected tumors in (F), as indicated. All data are presented as the mean ± SD (n ≥ 3). The *p*‐value in panels (C) and (G) were calculated using *one‐way ANOVA*. The *p*‐value in panels and (H) was calculated using *two‐way ANOVA*. ^***^ *p* < 0.001; ^****^ *p* < 0.0001.

Although FDX1 plays a crucial role in mediating cuproptosis, its biological function in breast cancer remains largely unelucidated. Our initial analysis indicated that TNBC patients with low FDX1 expression had worse prognosis (Figure S5A, Supporting Information). Further examination of FDX1 expression in the TNBC patients showed significantly higher expression levels in normal tissues than those in tumor tissues, both in the TCGA database and the database of our center (Figure S5B,C,E, Supporting Information). Consistent with these findings, TNBC cells displayed reduced FDX1 expression compared with normal breast cells (Figure S5D, Supporting Information). Knocking down *FDX1* enhanced the proliferative and colony‐forming abilities of triple‐negative breast cancer cells (Figure S5F–K, Supporting Information). To further dissect the biological function of AKT1‐mediated FDX1 phosphorylation, we introduced various FDX1 mutants (WT, S63A, and S63D) into TNBC cells (Figure S4D,E, Supporting Information). While the WT and S63A mutant FDX1 suppressed tumor progression, the S63D mutant substantially enhanced tumor growth (Figure 3I–L). Similar observations were obtained in xenograft mouse models, where the S63D mutant accelerated tumor growth and elevated the levels of the proliferation marker Ki67 (**Figure** **4**F–I). These results suggested that AKT1‐mediated FDX1 phosphorylation contributes to TNBC tumor growth.

Based on these findings, we hypothesized that AKT1 negatively regulated cuproptosis by phosphorylating FDX1. Cuproptosis relies on the lipoylation pathway and disruption of Fe‐S cluster biogenesis. To explore the relationship between phosphorylated FDX1 and Fe‐S cluster biogenesis, we examined the expression of Fe‐S cluster‐containing proteins. The FDX1‐S63D mutation did not affect the protein levels of POLD1, ACO2, SDHB, or DPYD, and a similar pattern was observed in cells overexpressing FDX1‐WT or FDX1‐S63A mutants (Figure 4A). Therefore, neither FDX1‐WT nor its phosphorylated form affected the synthesis of Fe‐S clusters, which was consistent with previous reports (Figure 4A).^[^ ^22^ ^]^ FDX1 acts as an upstream regulator of protein lipoylation, specifically affecting TCA cycle enzymes, including DLAT. Our observations revealed that the FDX1‐S63D mutant did not interact with DLAT (Figure S6G, Supporting Information). The FDX1‐S63D mutant showed a significant lipoylation defect in both DLAT and DLST, in contrast to FDX1‐WT (Figure 4B). Notably, compared to the WT or S63A mutant, the FDX1‐S63D mutant impaired cuproptosis in TNBC, as evidenced by reduced lipoylation and oligomerization of DLAT (Figure 4C–E; Figure S6H, Supporting Information). Next, we assessed the effect of FDX1‐WT and FDX1‐S63D overexpression cuproptosis sensitivity. FDX1‐WT increased TNBC cell sensitivity to cuproptosis, whereas the S63D mutation conferred resistance to copper ions (Figure S6A–F, Supporting Information). Consistently, in vivo studies demonstrated that the ectopic expression of the S63D mutant conferred resistance to elesclomol (Figure 4F–H). IHC staining confirmed that the S63D mutation impaired copper‐mediated DLAT lipoylation (Figure 4I). In summary, these findings suggested that AKT1‐mediated FDX1 phosphorylation contributed to cuproptosis resistance in TNBC cells.

### 2.5 AKT1‐Mediated FDX1 Phosphorylation Promotes TNBC Glycolysis

**Figure 5:**
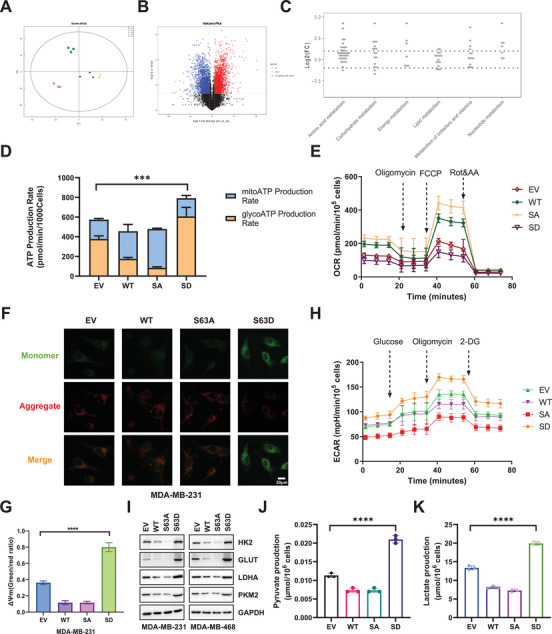
AKT1‐mediated FDX1 phosphorylation promotes glycolysis. A) MDA‐MB‐231 cells stably overexpressing FDX1 WT or variants were extracted for metabolomics analysis. Metabolites were visualized using principal component analysis. B) Differential metabolites between FDX1 S63A and S63D mutants were visualized using a volcano plot. C) Differential metabolites were subjected to KEGG pathway enrichment analysis. D) The real‐time ATP production rates of MDA‐MB‐231 cells overexpressing FDX1 S63A and S63D were measured following sequential injections of oligomycin and Rot/AA using the Seahorse XFe96 Analyzer (Agilent), *n* = 3. E) OCR responses of MDA‐MB‐231 cells stably overexpressing FDX1 WT or its variants were determined following sequential injections of oligomycin, trifluoromethoxy carbonylcyanide phenylhydrazone (FCCP), and rotenone, *n* = 8. F) Changes in mitochondrial membrane potential (ΔΨm) in MDA‐MB‐231 cells stably overexpressing FDX1 WT or its variants were evaluated using JC‐1 fluorescence. G) Fluorescence intensity was measured and quantified using ImageJ, and the red/green fluorescence ratio was calculated, *n* = 3. H) ECAR responses of MDA‐MB‐231 cells stably overexpressing FDX1 WT or its variants were measured following sequential injections of glucose, oligomycin, and 2‐deoxy‐D‐glucose (2DG), *n* = 8. I) IB analysis of MDA‐MB‐231 cells stably overexpressing FDX1 WT or its variants targeting key enzymes in the glycolytic pathway. J) Pyruvate production in MDA‐MB‐231 cells overexpressing FDX1 WT or its variants was measured, *n* = 3. K) Lactate production in MDA‐MB‐231 cells overexpressing FDX1 WT or its variants was measured, *n* = 3. All data are presented as the mean ± SD (n ≥ 3). The *p*‐values in panels (D), (G), (J) and (K) were calculated using *one‐way ANOVA*. ^***^ *p* < 0.001; ^****^ *p* < 0.0001.

Cuproptosis interacts with components of the TCA cycle in the mitochondria, whereas FDX1 acts upstream of the lipoylation pathway, which is crucial for respiratory chain function.^[^ ^22^ ^]^ Therefore, AKT1‐mediated FDX1 phosphorylation may also influence tumor metabolic reprogramming. Metabolomic analysis revealed that FDX1‐WT and S63A mutants induced similar changes in metabolites, whereas the S63D mutation exhibited notable differences, indicating distinct metabolic profiles (**Figure** **5**A). Further analysis of metabolite differences between the FDX1‐S63A and S63D mutants showed that the FDX1‐S63D mutant significantly promoted glucose, amino acid, and energy metabolism, favoring tumor malignant tumor proliferation and adaptation to adverse survival environments (Figure 5B,C). Moreover, the total ATP production rate in cells with the FDX1‐S63D mutation was significantly increased, primarily due to the activation of glycolysis, whereas mitochondrial ATP production was suppressed (Figure 5D). The oxygen consumption rate (OCR) assay results further indicated that phosphorylated FDX1 inhibited OCR, whereas FDX1‐WT and the FDX1‐S63A mutant enhanced OCR (Figure 5E). These findings suggested that phosphorylated FDX1 impaired mitochondrial function. To test this hypothesis, we assessed mitochondrial membrane potential using the JC‐1 dye assay and measured the red‐to‐green fluorescence ratio. The results showed that MDA‐MB‐231 cells transfected with FDX1‐S63A mutant exhibited strong red fluorescence, indicating increased mitochondrial membrane potential due to JC‐1 aggregation. Conversely, cells transfected with FDX1‐S63D mutant showed an elevated green‐to‐red fluorescence ratio (Figure 5F,G), suggesting mitochondrial dysfunction.

Tumor cells shift their energy metabolism toward glycolysis because of impaired aerobic respiration.^[^ ^31^, ^32^, ^33^ ^]^ Our findings demonstrated that phosphorylated FDX1 increased the extracellular acidification rate (ECAR), whereas FDX1‐WT or the FDX1‐S63A mutations resulted in a lower ECAR (Figure 5H). Phosphorylated FDX1 also upregulated several glycolysis‐related genes, including glucose transporter 1 (GLUT1), hexokinase 2 (HK2), pyruvate kinase M2 (PKM2), and lactate dehydrogenase A (LDHA), which ultimately promoted the glycolytic pathway (Figure 5I). Increased lactate and pyruvate production were observed in the FDX1‐S63D mutant, further indicating the pivotal role of the FDX1‐S63D mutation in promoting TNBC glycolysis (Figure 5J,K). Taken together, these data showed that phosphorylated FDX1 promoted the transition from mitochondrial respiration to glycolysis, allowing tumor cells to adapt to rapid growth.

### 2.6 Combination of AKT1 Inhibitor with Copper ion Carriers Effectively Inhibits TNBC Progression

**Figure 6:**
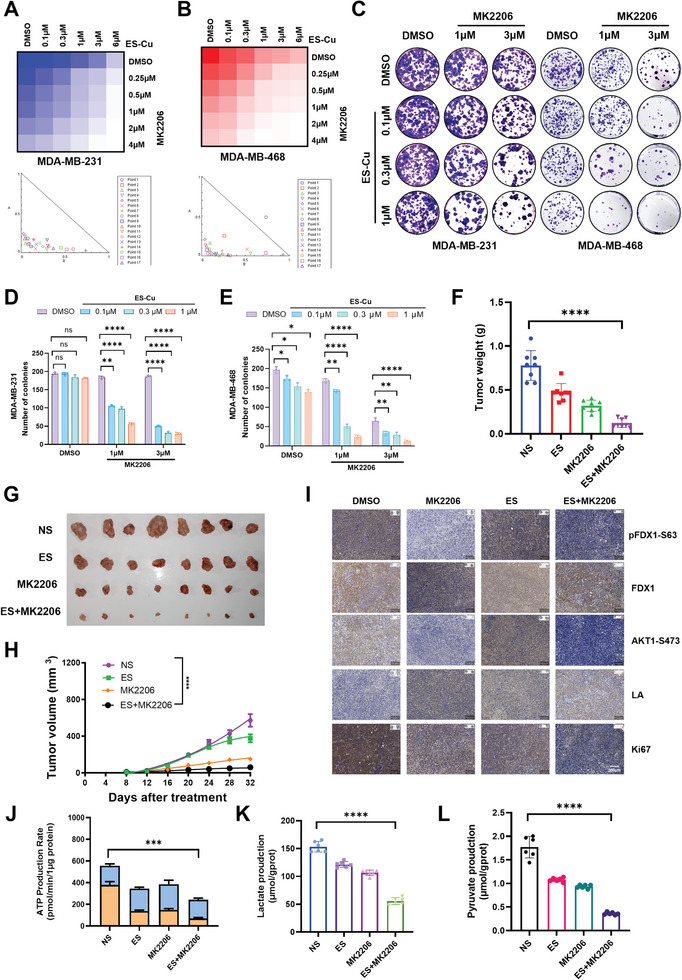
AKT1 inhibitors synergize with elesclomol in TNBC therapies. A) MDA‐MB‐231 cells were treated with DMSO, MK2206, elesclomol, or a combination of MK2206 and elesclomol‐Cu as indicated for 2 days and then subjected to a cell viability assay (top panel). The combination index (CI) was calculated (bottom panel), *n* = 3. B) MDA‐MB‐468 cells were treated with DMSO, MK2206, elesclomol, or a combination of MK2206 and elesclomol as indicated for 2 days and then subjected to a cell viability assay (top panel), *n* = 3. The combination index (CI) was calculated (bottom panel). C) MDA‐MB‐231 and MDA‐MB‐468 cells treated with MK2206, elesclomol, or a combination of MK2206 and elesclomol as indicated for 10 days were subjected to colony formation assays. D, E) Bar graphs provided a statistical evaluation of colony counts for MDA‐MB‐231 cells (D) and MDA‐MB‐468 cells (E), *n* = 3. F, G, H) MDA‐MB‐231 cells were subjected to mouse xenograft assays. Mice were administered vehicle, MK2206 (20 mg kg^−1^), elesclomol (20 mg kg^−1^), or a combination of MK2206 (20 mg kg^−1^) and elesclomol (20 mg kg^−1^) three times a week for 24 days (G). Dissected tumors were weighed (F), and tumor sizes were monitored (H), *n* = 8. I) IHC staining was performed on tissues derived from dissected tumors in (G), as indicated. J) The real‐time ATP production rates of cells extracted from tumor tissues generated in (G) were measured following sequential injections of oligomycin and Rot/AA using the Seahorse XFe96 Analyzer (Agilent), *n* = 3. K) Pyruvate production in cells extracted from tumor tissues generated in (G) was measured, *n* = 6. L) Lactate production in cells extracted from tumor tissues generated in (G) was measured, *n* = 6. All data are presented as the mean ± SD (n ≥ 3). The *p*‐value in panels (G), (J), (K) and (L) was calculated using *one‐way ANOVA*. The *p*‐values in panels (D), (E) and (F) were calculated using *two‐way ANOVA*. ^*^ *p* < 0.05; ^**^< 0.01; ^***^ *p* < 0.001; ^****^ *p* < 0.0001; ns, not significance. [Correction added on 26 February 2025, after first online publication: figure 6 is updated].

Our previous study demonstrated that AKT1‐mediated FDX1 phosphorylation inhibited cuproptosis and promoted TNBC progression. We observed a positive correlation between AKT1 activation, FDX1 phosphorylation at residue S63, and copper concentration, whereas lipoic DLAT levels were negatively correlated, as measured by IHC and IF staining (Figure 3M; Figure S5C, Supporting Information). Next, we evaluated whether combining AKT1 inhibition with copper ion carriers could offer therapeutic benefits for TNBC. The combination of MK2206, an AKT1 inhibitor, with copper ion carriers, elesclomol, and copper ions showed a strong synergistic effect with a combination index CI<1 in TNBC cells (**Figure** **6**A,B) and significantly suppressed colony formation (Figure 6C–E).

**Figure 7:**
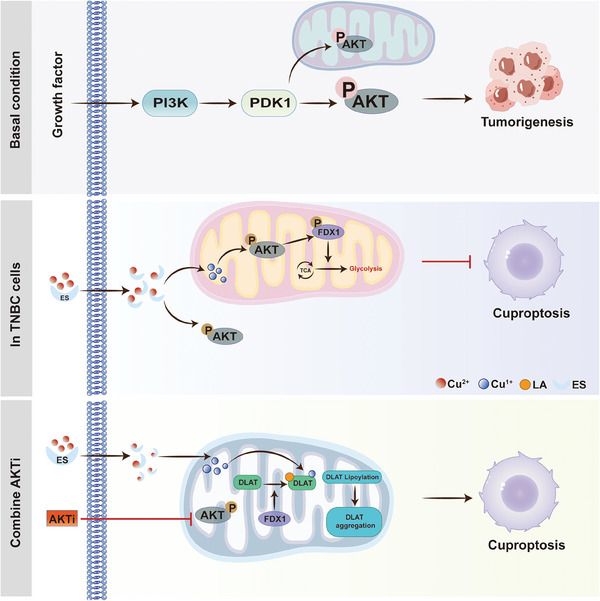
Model of AKT1 regulating cuproptosis in TNBC tumorigenesis. Schematic illustration of AKT1 role in regulating cuproptosis. Copper activates AKT signaling pathway, which inhibits cuproptosis by directly phosphorylating ferredoxin‐1 (FDX1), a key regulator of cuproptosis. AKT‐mediated FDX1 phosphorylation not only abrogates FDX1‐induced cuproptosis and aerobic respiration, but also promotes glycolysis. Consequently, combination of the AKT inhibitor MK2206 and the copper ionophore elesclomol synergistically induces cuproptosis and inhibits glycolysis, suggesting a feasible and effective therapeutic strategy for TNBC.

The combination treatment markedly inhibited tumor growth compared to treatment with either agent alone in vivo (Figure 6F–H). IHC staining further confirmed enhanced cuproptosis in TNBC tissues treated with MK2206 and elesclomol (Figure 6I). To explore the metabolic effects of this combination therapy, we assessed the ATP production pathways in tumor tissues. Combination treatment significantly reduced glycolysis‐driven ATP production (glycoATP) without affecting mitochondrial ATP (mitoATP) levels (Figure 6J). Moreover, lactate and pyruvate production notably decreased under the combination treatment (Figure 6K,L), indicating inhibition of glycolysis. In summary, these findings indicated that the combination of MK2206 and elesclomol effectively targeted this pathway and represented a promising therapeutic strategy for TNBC (**Figure** **7**).

## 3 Discussion

Cuproptosis is a form of mitochondrial cell death induced by proteotoxic stress. However, the regulatory mechanism in TNBC remains still unclear. In our study, we observed that copper overload resulted in abnormal activation of AKT1 in TNBC. AKT activation has been widely exploited in response to extracellular stimuli, including insulin, hypoxia, and high‐fat diet,^[^ ^34^, ^35^ ^]^ contributing to tumorigenesis and anti‐apoptosis effects. However, the roles of AKT in regulating cuproptosis remain undefined. Here, AKT1 has been characterized to directly phosphorylate FDX1 and inhibit aerobic respiration and cuproptosis. Furthermore, phosphorylated FDX1 shifts the cellular metabolism toward glycolysis, facilitating a rapid energy supply. Notably, the combination of the AKT1 inhibitor MK2206 and the copper ionophore elesclomol (ES) synergistically inhibit TNBC tumorigenesis both in vitro and in vivo.

Copper is an essential trace element that plays a pivotal role as a cofactor in biological redox reactions. It is extensively involved in biosynthesis within the human body and is tightly regulated by the coordinated activities of copper uptake by CTR1 and efflux by ATPases under normal physiological conditions.^[^ ^26^, ^36^, ^37^, ^38^ ^]^ The disruption of copper homeostasis can lead to disease development. In several solid tumors, including TNBC, copper levels in cancer tissues are higher than those in adjacent normal tissues, which correlates with advanced TNM stages, poor prognosis, and increased recurrence rates, making it a potential biomarker for cancer progression.^[^ ^10^, ^39^, ^40^ ^]^ Dysregulated copper metabolism plays a dual role in tumorigenesis and cancer therapy.^[^ ^41^ ^]^ Cuproplasia refers to copper‐dependent cell growth and proliferation, encompassing processes such as hyperplasia, metaplasia, and neoplasia through signaling pathways modulated by both enzymatic and non‐enzymatic copper.^[^ ^2^, ^41^ ^]^ More recently, copper treatment of cell cultures increased the phosphorylation of tyrosine kinases such as TRKB, EGFR, and MET, which are upstream regulators of the MAPK signaling pathway.^[^ ^42^ ^]^ Copper can also directly activate MEK, enhancing ERK phosphorylation and leading to MAPK pathway activation, which is a key driver of tumor cell growth and division.^[^ ^43^ ^]^ Additionally, copper promotes angiogenesis by transiently upregulating VEGF expression and activity, presumably through the generation of ROS.^[^ ^44^, ^45^ ^]^ Conversely, excess copper exerts cytotoxic effects on tumor cells by triggering cell death, particularly apoptosis, paraptosis, pyroptosis, ferroptosis, and cuproptosis. Copper overloads generate ROS, causing oxidative stress and damage to cellular components.^[^ ^46^, ^47^ ^]^ It disrupts mitochondrial function, impairs energy production and promotes apoptosis.^[^ ^48^ ^]^ Furthermore, copper induces ferroptosis by perturbing lipid peroxidation pathways.^[^ ^17^, ^49^ ^]^ It was recently reported that copper triggers cuproptosis by binding to lipoylated DLAT, a recently discovered cell death mechanism that disrupts the TCA cycle and leads to DLAT aggregation.^[^ ^21^ ^]^

The recent discovery of cuproptosis has promoted the targeting of copper metabolism in cancer cells. Copper promotes METTL16 lactylation at site K229 regulated by SIRT2 and subsequently mediates m6A modification of FDX1, followed by cuproptosis. Thus, combining elesclomol with the SIRT2‐specific inhibitor AGK2 induces cuproptosis in gastric tumors both in vitro and in vivo.^[^ ^24^ ^]^ MELK enhances the expression of the cuproptosis‐related signature gene DLAT, particularly by increasing the DLAT monomer portion through the activation of the PI3K/mTOR pathway, thereby facilitating the progression of HCC.^[^ ^50^ ^]^ In our study, we found that the copper ion content was significantly elevated in TNBC tissues and was closely associated with prognosis. However, TNBC cells are insensitive to cuproptosis. To investigate the mechanism contributing to TNBC resistance to cuproptosis, we performed a phosphoproteomic analysis and identified AKT1 as a key factor.

Copper is closely related to the AKT1 signaling pathway. Our findings demonstrated that copper/ES directly induced AKT1 phosphorylation in TNBC cells. Specifically, elesclomol and copper ions enhanced the interaction between AKT1 and PDK1, and knocking out PDK1 abolished the ability of elesclomol and copper ions to activate AKT1, which is consistent with the results of previous studies.^[^ ^29^, ^51^ ^]^ The AKT1 signaling pathway is a crucial oncogenic pathway that is abnormally activated in TNBC. Our previous study revealed that AKT1 phosphorylated SIK1, thereby disrupting its tumor‐suppressive function.^[^ ^52^ ^]^ Moreover, AKT1 is also closely associated with tumor tolerance to cell death.^[^ ^53^ ^]^ AKT1 directly phosphorylates key apoptotic regulators such as BAD, Caspase‐9, and BIM, thereby inhibiting tumor apoptosis.^[^ ^54^ ^]^ In addition, AKT1 directly phosphorylates TRPML1 at Ser343, and prevents its degradation of TRPML1, which leads to ferroptosis inhibition.^[^ ^55^ ^]^ In this study, we found that AKT1 inhibited cuproptosis in TNBC by phosphorylating FDX1. Thus, when copper ions are overloaded in TNBC, the excess copper paradoxically activates AKT1, ultimately suppressing cuproptosis.

Cuproptosis depends on the lipoylation pathway and the destabilization of Fe‐S cluster proteins. Neither WT nor phosphorylated FDX1 affected Fe‐S cluster biosynthesis, consistent with the results of previous studies showing that FDX2, rather than FDX1, is essential for Fe‐S cluster biosynthesis.^[^ ^23^ ^]^ FDX1 plays a pivotal role in cuproptosis by reducing Cu (II) to Cu (I) in the mitochondria, thereby promoting excessive protein lipoylation and subsequent aggregation, which are closely tied to mitochondrial respiration.^[^ ^56^ ^]^ In our study, we observed that the FDX1‐S63D mutant caused a significant DLAT lipoylation defect compared to FDX1‐WT. FDX1‐mediated lipoylation pathway is closely associated with aerobic respiration and cellular metabolism.^[^ ^41^ ^]^ FDX1‐knockout cells exhibit a failure of oxidative phosphorylation and accumulation of Kennedy pathway intermediates. We found that phosphorylated FDX1 induced mitochondrial damage, as evidenced by a decrease in mitochondrial membrane potential and OCR. Additionally, phosphorylated FDX1 promoted the expression of glycolysis‐related genes, thereby facilitating glycolysis. Phosphorylated FDX1 stimulated lactate and pyruvate production, thereby promoting rapid energy metabolism. Glycolysis status determines the susceptibility to cuproptosis. Clinical studies have revealed that patients with low lactate levels exhibit an increased sensitivity to elesclomol.^[^ ^30^, ^57^ ^]^ Therefore, inhibiting glycolysis enhances cuproptosis‐mediated suppression of tumor growth. AMPK increases the sensitivity of human pancreatic cancer cells to cuproptosis, owing to AMPK‐induced suppression of glycolysis.^[^ ^58^ ^]^ Similarly, ACOD1 enhances the susceptibility of CRC cells to cuproptosis by inhibiting aerobic glycolysis.^[^ ^59^ ^]^ Our findings revealed that the AKT1 inhibitor, MK2206, inhibited glycolysis in TNBC cells, thus, the combination of elesclomol with the AKT1 inhibitor has been shown to synergistically reduce cell viability.

Collectively, we reported that copper ion levels are elevated levels in TNBC cells, leading to AKT1 activation via PDK1. Nevertheless, AKT1 suppresses cuproptosis by phosphorylating FDX1, which explains the resistance of TNBC cells to cuproptosis. Phosphorylated FDX1 impairs mitochondrial function, inhibits aerobic respiration, and promotes glycolysis. Therefore, we propose a novel therapeutic strategy involving the synergistic combination of AKT1 inhibitors and copper ionophores to treat TNBC.

## 4 Experimental Section

### Cell Lines, Transfection, and INFECTION

MDA‐MB‐231, MDA‐MB‐468, and HEK293T cell lines were cultured in DMEM medium supplemented with 10% FBS. All cell lines were maintained at 37 °C in a humidified atmosphere with 5% CO_2_. Cell transfection was performed using Lipofectamine or PEI (Polysciences) according to the manufacturer's instructions. Packaging of lentiviral shRNA or over‐expression viruses, as well as subsequent infection of various cell lines were performed according to the methods described previously. After viral infection, cells maintain continuous screening with different concentrations of hygromycin or puromycin based on the cell line and the viral vector used to infect the cells.

### Antibodies and Reagents

Anti‐AKT Substrate (RxRxxpS/T) antibody (9614), anti‐phospho‐Ser473‐AKT antibody (4060), anti‐phospho‐Thr308‐AKT antibody (2965), anti‐AKT total antibody (4691), anti‐phospho‐Ser9‐GSK3β antibody (5558), anti‐GSK3β antibody (12456), anti‐PRAS40 antibody (2691), anti‐phospho‐PRAS40 (Thr246) antibody (2997), anti‐DLST antibody (11954), anti‐GST antibody (2622) and anti‐HA antibody (2367) were obtained from Cell Signaling Technology. Anti‐FDX1 antibody (12592‐1‐AP), anti‐DLAT antibody (13426‐1‐AP), anti‐GAPDH antibody (10494‐1‐AP) and anti‐AKT antibody (60203‐2‐Ig) were obtained from Proteintech. Anti‐LA antibody (ab58724) was obtained from Abcam. Anti‐Aconitase 2 antibody (A4524), anti‐SDHB antibody (A23832), anti‐POLD1 (A4218), anti‐DPYD (A1620), anti‐PDK1 (A8930) were obtained from ABclonal. AKT inhibitor MK2206 (HY‐108232), capivasertib (HY‐15431), copper ionophore elesclomol (HY‐12040), disulfiram (HY‐B0240), and insulin (HY‐P73243) were obtained from MCE.

### DNA Constructs and Mutant

pcDNA3‐HA‐myr‐AKT1, pcDNA3‐HA‐myr‐AKT2, pcDNA3‐HA‐myr‐AKT3, pcDNA3‐HA‐S6K1‐R3A, pcDNA3‐HA‐Δ60‐SGK1, pcDNA3‐HA‐AKT1 were cloned into mammalian expression pCDNA3‐HA vectors. pCMV‐GST‐FDX1 was cloned into mammalian expression pCMV‐GST vectors. pGEX‐4T1‐FDX1 were cloned into mammalian expression pGEX‐4T1 vectors. pLenti‐hygro‐FDX1 was cloned into mammalian expression pLenti‐hygro vectors. FDX1‐S63A, FDX1‐S63D, and AKT1‐E17K were obtained via Q5 Site‐Directed Mutagenesis Kit (New England Biolabs) according to the manufacturer's instructions and the sequences were verified by DNA sequencing. Specific shRNA primers were cloned into pLKO.1‐puro lentiviral vectors, human AKT1 target sequence #1 5′‐CGTGCCATGATCTGTATTTAA, human AKT1 target sequence #2 5′‐GGACAAGGACGGGCACATTAA, human AKT1 target sequence #3 5′‐CGCGTGACCATGAACGAGTTT, human FDX1 target sequence #1 5′‐GATGCCAGACAATCCATTGAT, human FDX1 target sequence #2 5′‐GCAATCACTGATGAGGAGAAT, human FDX1 target sequence #3 5′‐GCTGCCAAATCTGTTTGACAA.

### Breast Cancer Tissues and Immunohistochemical Staining

All breast cancer and paired normal specimens were obtained from the Women and Children's Medical Center of Guangzhou Medical University and usage of these specimens was approved by the institutional ethics committee (2023, No. 306B00). Informed consent was obtained from each patient. Fix tumor xenografts or surgical specimen tissue sections in 10% neutral buffered formalin, embedded in paraffin, and cut into 4 µm sections. For IHC staining, the sections were incubated with 3% H_2_O_2_ for 15min at room temperature to eliminate the endogenous peroxidase activity and blocked with 5% normal goat serum for 1 hr at room temperature. Sections were incubated with primary FDX1, FDX1‐pS63, AKT1‐ pS473, LA, Ki67 antibody at 4 °C overnight, followed by incubation with monoclonal mouse anti‐rabbit immunoglobulins (Dako#M0737) for 1 h at room temperature. Afterward, sections were incubated with Envision+ System‐HRP Labeled Polymer Anti‐Rabbit (Dako #K4003) for 30 min Specific detection was developed using the DAB chromogen kit (Dako#K3468) and lightly counterstained with hematoxylin.

### Copper Microplate Assay

The Copper (Cu) Colorimetric Assay Kit was purchased from Elabscience (E‐BC‐K300‐M). It measures the absorption or transmission of light by a copper‐containing solution. By analyzing the resulting spectra, the concentration of copper ions can be determined.

### Immunoprecipitation (IP), GST Pull‐Down (PD) Assay and Western Blot

Cells were lysed in EBC buffer (50 mm Tris pH 7.5, 120 mm NaCl, 0.5% NP‐40) supplemented with protease inhibitors (phosphatase inhibitor cocktail set I and II, Calbiochem) and phosphatase inhibitors (PhosSTOP, Roche). The protein concentrations of whole cell lysates were quantified by a bicinchoninic acid (BCA) assay kit (Pierce BCA Protein Assay Kit, 23225). An equal amount of whole cell lysates was separated by 10% SDS‐PAGE gels and then transferred to an Immobilon‐P PVDF membrane (Millipore, Massachusetts, USA). The membrane was incubated overnight with a primary antibody at 4 °C overnight and then hybridized with a secondary antibody at room temperature for 1h. The signals were visualized by enhanced chemiluminescence as previously described. For immunoprecipitation, WCL were incubated with the indicated antibody (1–2 µg) for 3–4 h at 4 °C followed by Protein A/G sepharose beads (GE Healthcare) at 4 °C overnight. For GST pull‐down assays, the lysates were incubated with glutathione sepharose 4B (GE Healthcare) for 2 hr at 4 °C. The immuno‐complexes were washed four times with NETN buffer (20 mm Tris, pH 8.0, 150 mm NaCl, 1 mM EDTA and 0.5% NP‐40) and then were resolved by SDS‐PAGE and immunoblotted with indicated antibodies. Quantification of the immunoblot band intensity was performed with ImageJ software.

### Soluble and Insoluble Fraction Isolation

Cells were harvested and lysed in ice‐cold NP40 lysis buffer (20 mm Tris (pH 7.4), 150 mm NaCl, 1% NP40) supplemented with protease inhibitors for 30 min. Then the cell extracts were centrifuged at 14 000 rpm at 4 °C for 30 min. Following centrifugation, the supernatant and pellet were carefully separated. The supernatant was mixed with 5× SDS loading buffer, while the pellet was resuspended in 1× SDS loading buffer. Two fractions were then boiled and sent for western blotting.

### Immunostaining (IF)

Cells grown on confocal dishes were fixed in paraformaldehyde, blocked with 10% goat serum, and then incubated with primary FDX1 and AKT antibodies, washed thrice with PBS, and then incubated with 488 Alexa (green) and 647 Alexa (red)‐labeled secondary antibodies. The DNA dye DAPI was used for re‐staining the nuclear DNA.

### Peptide Synthesis and Dot Immunoblot Assay

FDX1‐S63‐WT and FDX1‐S63‐Phospho peptides used for dot blot assays were synthesized by HuaBio Technology. The sequences are listed below:

FDX1‐S63‐WT: C‐SARARSSSEDKITVC

FDX1‐S63‐Phospho: C‐SARARSS(pS)EDKITVC

Peptides were diluted into 2 mg mL^−1^ for dot blot assays, and spotted onto the nitrocellulose membrane at doses of 0.01, 0.03, 0.10, and 0.30 mg. The membrane was dried and blocked by soaking in TBST buffer with 5% nonfat milk for immunoblot analysis.

### Purification of GST‐Tagged Proteins from Bacteria

Recombinant GST‐conjugated FDX1 was generated by transforming the BL21 (DE3) E. coli strain with pGEX‐ 4T1‐FDX1. Starter cultures grown overnight at 37 °C were injected (1%) into larger volumes. Cultures were grown at 37 °C to an O.D. 0.8, and then the protein expression was induced for 16 h using 0.1 mM isopropyl β‐d‐ 1‐thiogalactopyranoside (IPTG) at 16 °C with vigorous shaking.

Recombinant proteins were purified from harvested pellets, which were re‐suspended in EBC buffer for sonicated. Insoluble proteins and cell debris were discarded, and the supernatant was incubated with 50 µl 50% Glutathione‐sepharose slurry (Pierce) for 3 h at 4 °C and then washed 3 times with PBS buffe. Finnaly, the recombinant protein was stored at 4 °C in PBS buffer containing 10% glycerol or eluted by elution buffer. The purified proteins were analyzed by coomassie blue staining and quantified with BSA standards.

### In Vitro Kinase Assay

In vitro kinase assays were adapted from a protocol described previously. Briefly,1 µg of the bacterially purified GST‐FDX1 fusion proteins were incubated with immunoprecipitated AKT1 from cell lysates transfected in the presence of 200 µm adenosine triphosphate (ATP) in the kinase reaction buffer (50 mM Tris pH 7.5, 1 µm MnCl_2_, 2 mm DTT) for 30 min at 30 °C. The reaction was subsequently stopped by adding in 0.1 mm EDTA. The reaction was resolved by SDS‐PAGE and detected by pS63‐SIK1.

### Mass Spectrometry

For mass spectrometry (MS) analysis, GST‐pulldown were performed with the WCL derived from three 10 cm dishes of HEK293T cells transfected with GST‐FDX1 with/without HA‐myr‐AKT1 as previously described. The proteins were resolved by SDS‐PAGE and identified by Coomassie staining. The band containing FDX1 was reduced with 10 mm Dithiothreitol (DTT) for 30 min, alkylated with 55 mm iodoacetamide for 45 min, and in‐ gel‐digested with trypsin enzymes. The resulting peptides were extracted from the gel and analyzed by microcapillary reversed‐phase (C18) liquid chromatography‐tandem mass spectrometry (LC‐MS/MS), using a high resolution QExactive HF Orbitrap (Thermo Fisher Scientific) in positive ion DDA mode (Top 8) via higher energy collisional dissociation (HCD) coupled to a Proxeon EASY‐nLc II nano‐high performance liquid chromatography (HPLC). MS/MS data were searched against the Uniprot Human protein database (version 20151209 contain‐ ing 21,024 entries) using Mascot 2.5.1 (Matrix Science) and data analysis was performed using the Scaffold 4.4.8 software (Proteome Software). Peptides and modified peptides were accepted if they passed a 1% false discovery rate (FDR) threshold.

### Metabolomics

MDA‐MB‐231 cell stably overexpressing HA‐FDX1 WT or variants cultured for 48 h before washing with PBS. The samples were removed the protein by the precooled methanol/acetonitrile (1:1, v/v) and extracted the metabolites. The samples were then centrifuged at 14 000 g for 20 min at 4 °C to collect the supernatant. The supernatant was dried in a vacuum centrifuge. The samples were re‐dissolved in 100 µL acetonitrile/water (1:1, v/v) solvent and centrifuged at 14 000 g at 4 °C for 15 min, then the supernatant was injected for LC‐MS analysis. The mass spectrometry was performed using a UHPLC (1290 Infinity LC, Agilent Technologies) coupled to a quadrupole time‐of‐flight (AB Sciex TripleTOF 6600) and analyzed by NuoBiotech (Guangzhou)  Co., Ltd, China. The raw MS data were converted to MzXML files using ProteoWizard MSConvert before importing them into freely available XCMS software. For peak picking, the following parameters were used: centWave m/z = 10 ppm, peakwidth = c (10, 60), prefilter = c (10, 100). For peak grouping, bw = 5, mzwid = 0.025, minfrac = 0.5 were used. CAMERA (Collection of Algorithms of MEtabolite pRofile Annotation) was sued for annotation of isotopes and adducts. In the extracted ion features, only the variables having more than 50% of the nonzero measurement values in at least one group were kept. Compound identification of metabolites was performed by comparing of accuracy m/z value (<10 ppm), and MS/MS spectra with an in‐house database established with available authentic standards. After sum‐normalization, the processed data were analyzed by an R package (ropls), where it was subjected to multivariate data analysis, including Pareto‐scaled principal component analysis (PCA) and orthogonal partial least‐squares discriminant analysis (OPLS‐DA). The 7 fold cross‐validation and response permutation testing was used to evaluate the robustness of the model. The variable importance in the projection (VIP) value of each variable in the OPLS‐DA model was calculated to indicate its contribution to the classification. Student's *t*‐test was applied to determine the significance of differences between two groups of independent samples. VIP > 1 and *p*‐value < 0.05 were used to screen significant changed metabolites. Pearson's correlation analysis was performed to determine the correlation between two variables.

### Phosphoproteomic Profiling

The protein sample was extracted from WT and resistant MDA‐MB‐231 cell. The protein concentration was determined with the BCA kit according to the manufacturer's instructions. The protein solution was reduced with 5 mm dithiothreitol for 30 min at 56 °C and alkylated with 11 mm iodoacetamide for 15 min at room temperature in darkness. The protein sample was then diluted by adding 100 mm TEAB to urea concentration less than 2 m. Finally, trypsin was added at 1:50 trypsin‐to‐protein mass ratio for the first digestion overnight and 1:100 trypsin‐to‐protein mass ratio for a second 4 h‐digestion. Finally, the peptides were desalted by the C18 SPE column. Peptide mixtures were first incubated with IMAC microspheres suspension with vibration in loading buffer (50% acetonitrile/0.5% acetic acid). To remove the nonspecifically adsorbed peptides, the IMAC microspheres were washed with 50% acetonitrile/0.5% acetic acid and 30% acetonitrile/0.1% trifluoroacetic acid, sequentially. To elute the enriched phosphopeptides, the elution buffer containing 10% NH_4_OH was added and the enriched phosphopeptides were eluted with vibration. The supernatant containing phosphopeptides was collected and lyophilized for LC‐MS/MS analysis. The mass spectrometry was performed and analyzed by NuoBiotech (Guangzhou)  Co., Ltd, China. The resulting MS/MS data were processed using the MaxQuant search engine (v.1.6.15.0). Tandem mass spectra were searched against the Rattus_norvegicus_10116_PR_20230103.fasta (47945 entries) concatenated with reverse decoy database. Trypsin/P was specified as a cleavage enzyme, allowing up to 2 missing cleavages. The mass tolerance for precursor ions was set as 20 ppm in the first search and 20 ppm in the main search, and the mass tolerance for fragment ions was set as 0.02 Da. Carbamidomethyl on Cys was specified as fixed modification, and acetylation on protein N‐terminal and oxidation on Met and Phosphorylation modification were specified as variable modifications. FDR was adjusted to < 1%.

### Real‑time Cell Metabolism Assay

Real‐time ATP production, extracellular acidification rates (ECAR), and oxygen consumption rates (OCR) were quantified using Seahorse XFe96 and XFp Extracellular Flux Analyzers (Agilent, USA) with corresponding assay kits following the manufacturer's standardized protocols. MDA‐MB‐231 cells or dissected tumor tissues were seeded in appropriate Seahorse assay plates (96‐well or XFp) at a density of 1 × 10⁵ cells/well and prepared for assays by overnight culture or incubation to allow attachment. Before measurements, cells were washed, and the medium was replaced with Seahorse XF DMEM (pH 7.4) containing 10 mm glucose, 2 mm glutamine, and 1 mm sodium pyruvate. ATP production rates from mitochondrial respiration (mitoATP) and glycolysis (glycoATP) were recorded after sequential injections of 1.5 µm oligomycin and 0.5 µm rotenone/antimycin A (Rot/AA), while ECAR was quantified following injection of 2‐deoxy‐D‐glucose (2‐DG). Real‐time measurements of OCR were recorded under four sequential conditions: 1) basal respiration; 2) 1 µm oligomycin; 3) 0.3 µM FCCP; and 4) 0.5 µm rotenone and 0.5 µm antimycin. All OCR and ECAR values were normalized to the total protein content in each well.

### JC‐1 Staining

Treated MDA‐MB‐231 cells were seeded into confocal dishes and stained with the JC‐1 dye kit (Beyotime Biotechnology). After PBS washing, the JC‐1 staining solution was applied, and the cells were incubated at 37 °C for 30 min. Subsequently, images were captured with a Leica SP8 confocal microscope after washing with PBS. In healthy cells with a high mitochondrial membrane potential (△ψm), JC‐1 aggregates in the mitochondrial matrix, forming polymers that emit red fluorescence (Em = 590 nm). By contrast, mitochondria with reduced membrane potential retain JC‐1 in their monomeric form, emitting green fluorescence (Em = 529 nm). The green‐to‐red fluorescence intensity ratio was calculated as an indicator of △ψm.

### The Lactate and Pyruvate Production

MDA‐MB‐231 cells were seeded into six‐well plates at a density of 1 × 10^6^ cells per well and cultured overnight. The Cells or dissected tumor tissues were collected and extracted for further measurement. The lactate production and pyruvate production were detected using the Lactic Acid Content Assay Kit (Solarbio BC2235) and Pyruvate Content Assay Kit (Solarbio BC2205), according to the instruction of the manufacturers, respectively. All experiments were performed at least three times, and the data were normalized by the cell numbers or protein content.

### Colony Formation

Cells were inoculated into a 6‐well plate (300 or 600 cells/well) and cultured for 12–20 days until visible colonies were visible. The colonies were washed with PBS and fixed with 4% paraformaldehyde for 20 min and then stained with 0.4% crystal violet in 20% ethanol for 20 min. After staining, the plate was washed and air‐dried, and the number of colonies was numbered and quantified.

### Cell Proliferation Assay

The cells were seeded in the 96‐well plates (5000 cells/well). At the indicated time points, the cell proliferation was detected with MTS assay (Promega, G3530). The MTS reagent was added to cell culture media and incubated for 0.5–4 h in standard culture conditions. After incubation, the plate was shaken briefly and measured absorbance at 490 nm.

### Xenograft Models and In Vivo Treatments

Mouse xenograft assays were performed as described previously. All animals were approved by the Institutional Animal Care and Use Committee of Guangzhou Women and Children's Medical Center (RSDW‐2024‐01240) and purchased from Guangdong GemPharmatech. Briefly, 2 × 10^6^ MDA‐MB‐231 cells stably expressing targeting gene mixed with matrigel were injected into the flank of female nude mice (8/group, 4 weeks of age). Once the tumor size reached 50–100 mm^3^, some groups were treated with vehicles, MK2206 (20 mg kg^−1^), elesclomol (20 mg kg^−1^), or a combination of MK2206(20 mg kg^−1^)/elesclomol (20 mg kg^−1^) three times a week. Tumor size was measured every three days with a caliper, and the tumor volume was determined with the formula L × W^2^ × 0.5, where L is the longest diameter and W is the shortest diameter. Mice were euthanized when their tumor volume was larger than 2000 mm^3^.

### Statistical Analysis

The key experiments have been repeated at least 3 times, quantitated, and subjected to statistical analysis. GraphPad Prism version 10.0 was used for statistical analyses. As indicated in the figure legends, all quantitative data were presented as the mean & SD of three biologically independent experiments or samples; n and *p* values were indicated in every single figure. *Two‐way ANOVA* was used for multiple‐group comparisons, *one‐way ANOVA* for one‐way comparisons, and unpaired *two‐tailed t‐tests* for two‐group comparisons. Differences between groups were considered statistically significant at ^*^ *p* < 0.05, ^**^ *p* < 0.01, ^***^ *p* < 0.001, and ^****^ *p* < 0.0001; “ns” indicated not significant.

## Conflict of Interest

The authors declare no conflict of interest.

## Author Contributions

This study was conceived and designed by J.L. Development of methodology was performed by Z.S. and H.X. Acquisition of data (provided animals, acquired, and managed patients, provided facilities, etc.) was performed by Z.S., H.X., G.L., C.Y., X.G., J.Z., X.L., and Y.C. The final manuscript was written by Z.S, H.X., J.G., and J.L. Administrative, technical, or material support (i.e., reporting or organizing data, constructing databases) was provided by Z.S., H.X., K.W., J.G., and J.L. Study supervision was provided by J.L. All authors approved the manuscript.

## Supporting information



Supplemental information
