# Supplementary material for: AKT1 Phosphorylates FDX1 to Promote Cuproptosis Resistance in Triple‐Negative Breast Cancer
Source: Adv Sci (Weinh). 2025 Feb 20;12(17):2408106. doi: 10.1002/advs.202408106 (PMC12061301; doi:10.1002/advs.202408106)
Supplement: Supplementary file 1 — Supplemental information [file ADVS-12-2408106-s001.docx]

**AKT1 Phosphorylates FDX1 to Promote Cuproptosis Resistance in Triple-negative Breast Cancer**

Zicheng Sun^1#^, Huazhen Xu^1#^, Guanming Lu^2,3#^, Ciqiu Yang^4#^, Xinya Gao^1^, Jing Zhang^1^, Xin Liu^1^, Kun Wang^4#^, Jianping Guo^5, *^, Jie Li^1, *^,

Keywords: Cuproptosis; FDX1; AKT1; Metabolic reprogramming; Breast cancer

^1^ Department of Breast and Thyroid Surgery, Guangzhou Women and Children's Medical Center, Guangzhou Medical University, Guangdong 510000, China

^2^ Department of Breast and Thyroid Surgery, Affiliated Hospital of Youjiang Medical University for Nationalities, Guangxi 533000, China

^3^ Key Laloratory of Molecular Pathology in Tumors of Guangxi, Baise, Guangxi 533000, China

^4^ Department of Breast Cancer, Guangdong Provincial People’s Hospital (Guangdong Academy of Medical Sciences), Southern Medical University, Guangdong, 510000, China

^5^ Institute of Precision Medicine, The First Affiliated Hospital, Sun Yat-sen University, Guangdong 510000, China

^#^Corresponding Authors:

Guojp6@mail.sysu.edu.cn (J.G.)

Lijie2958@gwcmc.org (J.L.)

**Fig. S1. AKT1 is activated in elesclomol-resistant TNBC. A.** Morphology of WT and resistant MDA-MB-231 cells under elesclomol-Cu treatment for 24 hours. **B.** Bar graphs provided a statistical evaluation of the number of WT and resistant MDA-MB-231 cells under elesclomol-Cu treatment for 24 hours, n = 3. **C.** The lipoylated DLAT expression was analyzed in WT and resistant MDA-MB-231 cells treated with elesclomol for a 2-hour pulse. **D.** DLAT foci in WT and resistant MDA-MB-231 cells were imaged through immunofluorescence (DLAT: green, Mitotracker: red, DAPI: blue). **E.** Bar graphs provided a statistical evaluation of the number of DLAT foci, which were segmented and quantified using Image J, n = 3. **F.** DLAT oligomerization was analyzed in WT and resistant MDA-MB-231 cells treated with elesclomol for a 2-hour pulse. **G.** Differential proteins between WT and resistant MDA-MB-231 cells were subjected to KEGG pathway enrichment analysis. **H.** Differential proteins between WT and resistant MDA-MB-231 cells were subjected to domain enrichment analysis. **I.** IB analysis of WCL derived from WT and resistant MDA-MB-231 cells after different concentrations of elesclomol-Cu treatmen for 24 hours. **J.** IB analysis of WCL and immunoprecipitation products from HEK-293T cells transfected with Flag-PDK1 and HA-AKT1 after elesclomol-Cu treatment for 24 hours. **K.** IB analysis of WCL from MDA-MB-231 cells transfected with siRNA targeting PDK1 after elesclomol-Cu treatment. **L.** IB analysis of WCL from WT and resistant MDA-MB-231 cells. All data are presented as the mean ± SD (n ≥ 3). The p-value in panels (B) and (E) was calculated using one-way ANOVA. **<0.01; ***p < 0.001; ****p < 0.0001; ns, not significance.

**Fig. S2.** **AKT1 inhibits cuproptosis in triple-negative breast cancer. A.** MDA-MB-231 cells (top panel) and MDA-MB-468 cells (bottom panel) transduced with lentiviral shRNAs against AKT1 were treated with DMSO or disulfiram-Cu and subjected to CCK-8 cell viability assays, n = 3. **B.** MDA-MB-231 cells and MDA-MB-468 cells generated in (A) were treated with DMSO or disulfiram-Cu and subjected to colony formation assays. **C.** Bar graphs provided a statistical evaluation of colony counts for MDA-MB-231 cells (top panel) and MDA-MB-468 cells (bottom panel), n = 3. **D.** Lipoylated DLAT expression was analyzed in MDA-MB-231 cells with or without MK2206 treatment following a 2-hour elesclomol-Cu pulse. **E.** Lipoylated DLAT expression was analyzed in MDA-MB-468 cells with or without MK2206 treatment following a 2-hour elesclomol-Cu pulse. **F.** DLAT oligomerization was analyzed in MDA-MB-231 cells and MDA-MB-468 cells with or without MK2206 treatment following a 2-hour elesclomol-Cu pulse. **G.** DLAT foci in MDA-MB-231 cells with or without MK2206 treatment were imaged through immunofluorescence (DLAT: green, Mitotracker: red, DAPI: blue). **H.** Bar graphs provided a statistical evaluation of DLAT foci (G), which were segmented and quantified using ImageJ, n = 3. **I.** DLAT oligomerization was analyzed in MDA-MB-231 cells with or without insulin treatment following a 2-hour elesclomol-Cu pulse. **J.** DLAT oligomerization was analyzed in MDA-MB-468 cells with or without insulin treatment following a 2-hour elesclomol-Cu pulse. **K.** DLAT oligomerization was analyzed in MDA-MB-231 and MDA-MB-468 cells with or without insulin treatment following a 2-hour elesclomol-Cu pulse. All data are presented as the mean ± SD (n ≥ 3). The *p*-value in panel (A) and panel (C) was calculated using *two-way ANOVA*. The *p*-value in panel (H) was calculated using *one-way ANOVA*. **<0.01; ***p < 0.001; ****p < 0.0001.

**Fig. S3. AKT1 inhibits cuproptosis by targeting FDX1. A.** IB analysis of WCL and IP products derived from MDA-MB-231 cells. IgG was used as a negative control. **B.** IB analysis of WCL and GST-pulldown products derived from HEK-293T cells transfected with GST-FDX1 and HA-AKT1. **C.** In vitro protein binding analysis of FDX1 and AKT1. **D.** IB analysis of WCL and GST-pulldown products derived from HEK-293T cells transfected with GST-FDX1 truncations and HA-AKT1. **E.** In vitro protein binding analysis of FDX1 truncations (N-terminal, C-terminal) and AKT1. **F.** FDX1 and AKT1 staining was imaged through immunofluorescence in MDA-MB-231 and MDA-MB-468 cells.

**Fig. S4. AKT1 interacts with and phosphorylates FDX1. A.** IB analysis of WCL and GST-pulldown products derived from HEK-293T cells transfected with the indicated active AGC kinase and FDX1. **B.** IB analysis of WCL and GST-pulldown products derived from HEK-293T cells transfected with GST-FDX1 truncations and HA-myr-AKT1. **C.** In vitro kinase assays were performed using purified AKT1 proteins from HEK-293T cells transfected with HA-myr-AKT1 as the kinase source, and bacterially purified GST-FDX1 truncations as the substrate. **D.** IB analysis of WCL derived from MDA-MB-231 cells stably overexpressing HA-FDX1 WT or variants. **E.** IB analysis of WCL derived from MDA-MB-468 cells stably overexpressing HA-FDX1 WT or variants.

**Fig. S5. FDX1 plays tumor suppressor roles in breast cancer. A.** Kaplan-Meier analysis of the correlation between FDX1 expression and overall survival in breast cancer based on TCGA database. **B.** The relative expression levels of FDX1 in breast cancer based on TCGA database. **C.** IB analysis of WCL derived from breast cancer tissues, as indicated. **D.** IB analysis of WCL derived from normal mammary cell line (MCF-10A) and various breast cancer cell lines. **E.** IHC staining analysis of breast cancer tissues, as indicated. **F.** IB analysis of WCL derived from MDA-MB-231 cells transduced with lentiviral shRNA against FDX1. **G.** IB analysis of WCL derived from MDA-MB-468 cells transduced with lentiviral shRNA against FDX1. **H, I.** Cells generated in (F, G) were subjected to CCK-8-mediated cell viability assays, n = 3. **J, K.** Cells generated in (F, G) were subjected to colony formation assays, and the relative colony numbers were quantified in the bottom panel, n = 3. All data are presented as the mean ± SD (n ≥ 3). The *p*-value in panel (H) and (I) were calculated using *two-way ANOVA* t. The *p*-value in panel (J) and (K) were calculated using *one-way ANOVA*. ****p < 0.0001.

**Fig. S6.** **AKT1-mediated FDX1 phosphorylation inhibits cuproptosis. A.** MDA-MB-231 cells (top panel) and MDA-MB-468 cells (bottom panel) stably overexpressing FDX1 WT or variants were treated with elesclomol-Cu and subjected to CCK-8-mediated cell viability assays, n = 3. **B.** Cells generated in (A) were treated with elesclomol-Cu and subjected to colony formation assays. **C.** Bar graphs provided a statistical evaluation of the number of colonies for (B) MDA-MB-231 cells (left panel) and MDA-MB-468 cells (right panel), n = 3. **D.** MDA-MB-231 cells (top panel) and MDA-MB-468 cells (bottom panel) stably overexpressing FDX1 WT or variants were treated with DMSO or disulfiram-Cu subjected to CCK-8-mediated cell viability assays, n = 3. **E.** Cells generated in (A) were treated with disulfiram-Cu as indicated and subjected to colony formation assays. **F.** Bar graphs provided a statistical evaluation of the number of colonies for (E) MDA-MB-231 cells (left panel) and MDA-MB-468 cells (right panel), n = 3. **G.** IB analysis of WCL and HA immunoprecipitation derived from MDA-MB-231 cells stably overexpressing HA-FDX1 WT or variants. **H.** Lipoylated DLAT expression was analyzed in cells generated in (A) treated with elesclomol-Cu for a 2-hour pulse. All data are presented as the mean ± SD (n ≥ 3). The *p*-value in panel (A), (C), (D) and (F) were calculated using *two-way ANOVA*. The *p*-value in panel were calculated using *two-way ANOVA*. ****p < 0.0001.
